# *QuickStats*: Age-Adjusted Percentages* of Current Smokers^†^ Among Adults Aged ≥18 Years, by Sex, Race, and Hispanic Origin^§^ — National Health Interview Survey, 2016^¶^

**DOI:** 10.15585/mmwr.mm6701a10

**Published:** 2018-01-12

**Figure:**
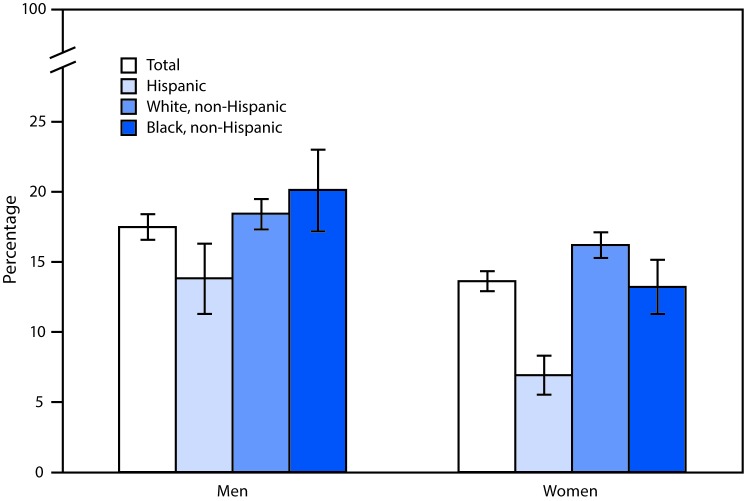
In 2016, men aged ≥18 years were more likely to be current smokers than women (17.5% compared with 13.6%). Non-Hispanic black men (20.1%) and non-Hispanic white men (18.4%) were more likely to be current smokers than Hispanic men (13.8%). Non-Hispanic white women (16.2%) were more likely to be current smokers than non-Hispanic black women (13.2%) and Hispanic women (6.9%).

**Source:** National Health Interview Survey, 2016. https://www.cdc.gov/nchs/nhis/index.htm.

